# Application of Imaging Indicators Based on ^18^F-Fluorodeoxyglucose Positron Emission Tomography/Computed Tomography in Colorectal Peritoneal Carcinomatosis

**DOI:** 10.3389/fonc.2022.888680

**Published:** 2022-05-26

**Authors:** Chun-Feng Sun, Ding Zhang, Yan Gao, Xiao-Ying Mao, Zhong-Hua Tan, Shan-Lei Bao, Chen Shen

**Affiliations:** ^1^ Department of Nuclear Medicine, Affiliated Hospital of Nantong University, Jiangsu, China; ^2^ Department of General Surgery, Affiliated Hospital of Nantong University, Jiangsu, China

**Keywords:** colorectal peritoneal carcinomatosis, colorectal cancer, peritoneal carcinomatosis, ^18^F-FDG, PET/CT, retention index, pathological type

## Abstract

**Objective:**

The imaging features of peritoneal carcinomatosis (PC) with different locations and pathological types of colorectal cancer (CRC) on ^18^F-fluorodeoxyglucose positron emission tomography/computed tomography (18F-FDG PET/CT) were analyzed and discussed.

**Methods:**

The PET/CT data of 132 patients with colorectal peritoneal carcinomatosis (CRPC) who met the inclusion and exclusion criteria between May 30, 2016, and December 31, 2019, were collected and analyzed. Observations included the location and pathological type of CRC, the peritoneal cancer index (PCI), standardized uptake maximum value (SUV_max_), and retention index (RI) of the CRPC. Statistical analysis was performed using SPSS 20.0 software, and *P* < 0.05 was considered statistically significant.

**Results:**

(1) The range of the PCI in the 132 patients studied was 2–30, with a mean value of 7.40 ± 8.14. The maximum long diameter of the CRPC lesions ranged from 0.6 to 12.1 cm, with an average of 3.23 ± 1.94 cm. The SUV_max_ ranged from 1.2 to 31.0, with a mean value of 9.65 ± 6.01. The SUV_max_ and size correlation coefficient for maximal CRPC lesions was r = 0.47 (*P* < 0.001). The RI range of the 72 patients who underwent time-lapse scanning was -10.0–112.2%, with RI quartiles of 13.5–48.9%; RI was ≥5% in 65 cases and <5% in seven cases. (2) The patients were grouped by the location of their CRC: the right-sided colon cancer (RCC, *n* = 37), left-sided colon cancer (LCC, *n* = 44), and rectal cancer groups (RC, *n* = 51). There were significant differences in the CRC pathological types (*P* = 0.009) and PCI scores (*P* = 0.02) between the RCC and RC groups and the RI between the RCC group and the other two groups (*P* < 0.001). (3) There were 88 patients organized into three groups by the pathology of their CRC: the moderately well-differentiated adenocarcinoma (group A, *n* = 57), poorly differentiated adenocarcinoma (group B, *n* = 16), and mucinous adenocarcinoma groups (group C, *n* = 15 cases, including one case of signet-ring cell carcinoma). There were significant differences in the CRC position (*P* = 0.003) and SUV_max_ (*P* = 0.03) between groups A and C.

**Conclusion:**

The PCI, SUV_max_, and RI of peritoneal metastatic carcinoma caused by CRC in different locations and pathological types vary. Mucinous adenocarcinoma and poorly differentiated adenocarcinoma are relatively common in the right colon, and the PCI of peritoneal metastatic carcinoma is fairly high, but the SUV_max_ and RI are somewhat low.

## Introduction

Colorectal cancer (CRC) can lead to peritoneal carcinomatosis (PC) through local dissemination and the implantation of metastases in the abdominal cavity. Colorectal peritoneal carcinomatosis (CRPC) is the second leading cause of death in patients with CRC. Given its importance in the clinical diagnosis and treatment process, the eighth edition of the cancer staging system released by the American Joint Committee on Cancer in 2018 (1) further refined metastasis (M) staging by adding a new M1c stage, i.e., CRPC with or without the combination of metastases from other organs or sites in the IV_C_ stage.

The clinical use of ^18^F-fluorodeoxyglucose (^18^F-FDG) positron emission tomography/computed tomography (PET/CT) in malignant tumors is becoming increasingly widespread, and its cost-effectiveness has been recognized in many ways (2, 3). The imaging evaluation of CRPC was previously based on CT or magnetic resonance imaging, but the scope of evaluation was limited, and false-negative results were common (4–7). In patients with CRC, ^18^F-FDG PET/CT has been found to be an effective way of assessing CRPC simultaneously (8–10).

In clinical practice, primary lesions and peritoneal metastases of colorectal mucinous adenocarcinoma generally show low density and glucose metabolism, which poses certain difficulties in imaging diagnosis and evaluation. Although the delayed imaging of ^18^F-FDG PET/CT is valuable for diagnosing some tumors (11, 12), can the delayed imaging of CRPC provide more valuable information? Is there any difference in peritoneal metastatic carcinoma caused by CRC in different locations and pathological types? A review of the literature on the correlation between the ^18^F-FDG PET/CT peritoneal cancer index (PCI), glucose metabolism index and the primary lesions of CRC found no previous relevant research. Therefore, this study aimed to analyze the imaging presentation of CRPC on ^18^F-FDG PET/CT and explore the relationship between the parameters of peritoneal cancer and the primary lesions of CRC.

## Materials and Methods

### Subjects

The data of 441 patients with CRC examined at the PET/CT Center of the Department of Nuclear Medicine of the Affiliated Hospital of Nantong University between May 30, 2016, and December 31, 2019, were retrospectively analyzed.

Inclusion criteria: (1) patients aged 18 to 85 years; (2) pathologically confirmed diagnosis of colorectal adenocarcinoma, mucinous adenocarcinoma, or signet-ring cell carcinoma; (3) clinical and imaging follow-up and/or pathological diagnosis of PC.

Exclusion criteria: (1) concurrent lesions of other malignant tumors, abdominal trauma, abdominopelvic infection, etc.; (2) patient had undergone chemotherapy, radiotherapy, targeted therapy, or immunotherapy within three months prior to the examination; (3) patient had peritoneal lesions that could not be characterized or was lost to follow-up.

**Figure 1:**
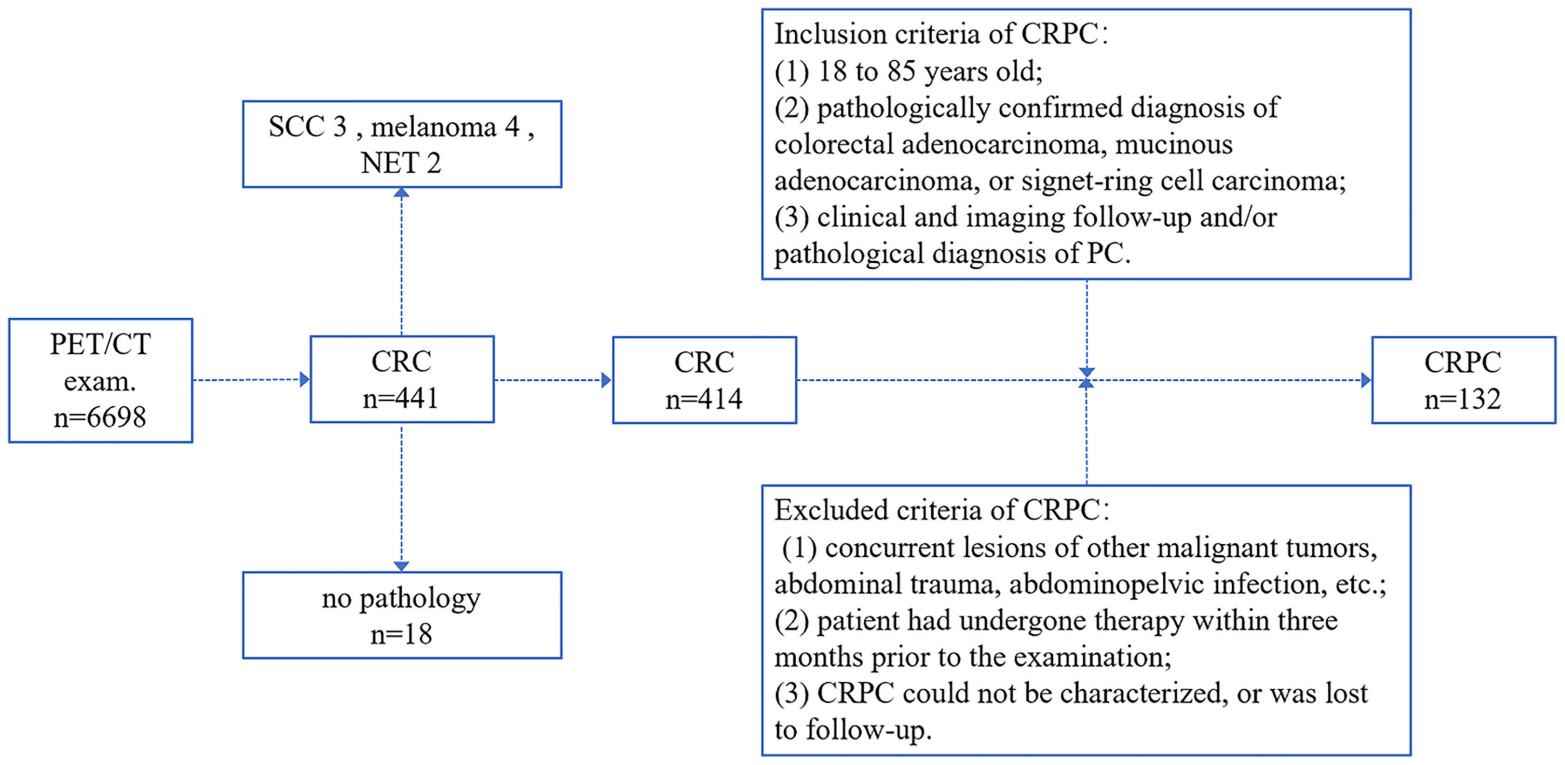
The colorectal peritoneal carcinomatosis (CRPC) patient inclusion process. CRC, colorectal cancer; PC, peritoneal carcinomatosis; SCC, squamous cell carcinoma; NET, neuroendocrine tumor.

Colorectal peritoneal carcinomatosis (CRPC) occurred in 132 of the 441 patients diagnosed with CRC (i.e., 132 patients met the inclusion and exclusion criteria), representing approximately 29.9% of the sample. The enrollment process is shown in **Figure 1**. The mean age of the 132 patients with CRPC was approximately 61.81 ± 11.52 years; 82 of these patients were male, with a mean age of 62.57 ± 11.51 years, and 50 were female, with a mean age of 61.59 ± 11.42 years. There was no statistical difference in age between the males and females (*t* = 0.48, *P* = 0.635). The primary CRC lesions were singles in 128 cases and multiples in four cases (classified as right or left colon according to the primary lesion).

The 132 cases were divided into three groups according to the location of the CRC: the right-sided colon cancer (RCC; *n* = 37), left-sided colon cancer (LCC; *n* = 44), and rectal cancer groups (RC; *n* = 51) using the right two-thirds and left one-third of the transverse colon as the boundary and location of the primary lesion in cases with multiple lesions. Of the 132 enrolled patients, 44 did not have surgery after gross pathological findings were obtained by colonoscopic biopsy (not included in the analysis by pathological type). The remaining 88 patients had surgical pathological findings and were divided into three groups according to their primary pathological type: the moderately well-differentiated (*n* = 57), poorly differentiated (*n* = 16), and mucinous adenocarcinoma groups (*n* = 15, including one case of signet-ring cell carcinoma).

The study was conducted in accordance with the Declaration of Helsinki and was approved by the Ethics Committee of the Affiliated Hospital of Nantong University. The patient’s informed consent was not required, as the study was a retrospective analysis of relevant data only and did not require direct patient participation.

### Positron Emission Tomography/Computed Tomography Imaging

The radiopharmaceutical ^18^F-FDG was provided by Nanjing Jiangyuan Andike Positron Research and Development Co., Ltd. (China) and required radiochemical purity >95%. All patients fasted for at least 6 h and had a blood glucose level below 150 mg/dl before the intravenous administration of ^18^F-FDG. Approximately 60 min after the injection of 4.07 MBq/kg of ^18^F-FDG, PET/CT scans of the enrolled patients were performed from the skull base to the proximal thigh using a 64-slice spiral CT scanner (GE Healthcare, Discovery™ 710, USA), followed by PET image acquisition in 3D mode. The CT scanning parameters were 120 kVp, 40–80 mA, display field of view (DFOV) 70.0 cm, helical thickness 3.75 mm, interval 3.27 mm, and matrix size 512 × 512. The PET scan was done in six to eight beds according to height, each 2–3 minutes, matrix size 192 × 192. Reconstruction was performed using the ordered subset expectation maximization. After the acquisition, the data were transferred to the AW workstation, and PET/CT image fusion and post-processing were performed using Advance Volume Share 5 (AW 4.6) software. The PET/CT scan was repeated 2 h after the injection of ^18^F-FDG under the same conditions as those chosen for the earlier scan.

### Image Analysis

The most commonly used international grading system for PC is Harmon and Sugarbaker’s proposed PCI grading system (13). Detailed results are shown in **Table 1**. In the present study, the size of the largest CRPC lesion (lesion size, LS) in each of these 13 regions was measured, and a composite score was assigned: LS-0 = no CRPC lesion; LS-1 = CRPC lesion <0.5 cm; LS-2 = CRPC lesion 0.5–5.0 cm; and LS-3 = CRPC lesion >5.0 cm. A composite PCI score of 0 referred to the absence of CRPC in a patient, and the maximum score of 39 indicated that a patient had a CRPC >5.0 cm in every region. Where the boundaries of a fusion lesion could be distinguished, it was recorded as two or more lesions; where the boundaries could not be distinguished, it was recorded as one lesion. Primary tumors and/or local recurrences that could be resected were not counted. Those fused with adjacent organs and could not be distinguished directly were scored as LS-3. The measurement of LS required a combination of PET and CT images to first identify the target lesion, with images showing the lesion border being measured preferentially on CT; otherwise, PET images were used (left–right or anterior–posterior diameters were included where possible to avoid respiratory motion effects). The largest CRPC lesions were counted based on the long diameter.

**Table 1:** The PCI grading system.

	Region	Anatomical region
Abdominopelvic regions	0	Umbilical region
	1	Right hypochondriac region
	2	Epigastric region
	3	Left hypochondriac region
	4	Lleft lumbar region
	5	Left inguinal region
	6	Lower abdominal region
	7	Right inguinal region
	8	Right lumbar region
Small intestinal regions	9	Upper jejunum
	10	Lower jejunum
	11	Upper ileum
	12	Llower ileum
Scoring based on LS	0	No CRPC lesion
	1	< 0.5 cm
	2	0.5–5.0 cm
	3	> 5.0 cm

LS, lesion size; This system divides the abdominal cavity into nine abdominopelvic regions (0-8 region) and four small intestinal regions (9-12 region), for a total of 13 regions. The size of the largest CRPC lesion in each of these 13 regions was measured, and a composite score was assigned based on LS. Each region is scored separately and accumulated to form the PCI score of this CRPC patient. The PCI score range for each CRPC patient was 0-39.

The standardized uptake value (SUV) was automatically calculated by a computer, and the maximum value (SUV_max_) was taken using the region of interest technique: SUV = mean localized area of interest activity (MBq/ml)/(injected activity [MBq]/body weight [g]) in g/ml. Patients undergoing a time-lapse examination were given a retention index (RI) based on the formula RI = ([SUV_max (time-lapse)_ – SUV_max (early)_]/SUV_max (early)_) × 100%, with RI ≥5% being positive and RI <5% being negative; RI ≥20% was significantly elevated, RI 5%–20% was elevated, and RI <5% was not significantly changed.

The image analysis and data measurements were performed by two experienced diagnosticians who had been practicing PET/CT for at least three years, and the quantitative values were measured by both physicians separately and averaged. In the event of a large difference between the results obtained by the two physicians and in case of disagreement, the decision was made by a higher-level physician. The SUV_max_ and RI of each patient with CRPC were obtained by analyzing the largest CRPC lesion.

### Colorectal Peritoneal Carcinomatosis Diagnosis

The diagnosis of CRPC was consistent with one of the following: (1) surgical pathological confirmation (including postoperative reoperation and laparoscopy); (2) pathological confirmation by puncture biopsy; (3) finding tumor cells on cytological examination of ascites; (4) imaging examination confirmed that the peritoneal lesions had shrunk or disappeared after treatment (on at least two types of imaging diagnoses or two instances of consistent imaging diagnosis); (5) imaging examination (at least two types of imaging diagnoses or two instances of consistent imaging diagnosis) confirmed progression of the lesion during follow-up (increase in size and diameter, increase in, or addition of, SUV_max_, etc.).

In this study, 132 cases of CRPC were diagnosed: 15 by surgical pathology, 32 by a pathological diagnosis of a lesion puncture biopsy, 13 by identifying exfoliated cells and cell blocks from ascites in immunohistochemistry, four by imaging follow-up of lesion improvement (after treatment), and 68 by imaging follow-up of lesion progression. For small CRPC lesions prone to partial volume effect, the following methods were adopted to assist analysis: (1) combined PET and CT; (2) thin-slice CT images; (3) time-lapse imaging; (4) multiple imaging examinations; (5) intertemporal dynamic imaging examination.

### Statistical Analysis

The data were entered and collated using Excel spreadsheets and analyzed using SPSS 20.0 statistical software. Statistical descriptions included means and standard deviations of continuous variables, numbers, and percentages of absolute quantities. An independent sample *t*-test was used to compare the means of two samples in the measurement data. A one-way analysis of variance (ANOVA) was used to compare the overall indicators of more than three groups, a least significant difference *post hoc* test was used for pairwise comparison, and a Games–Howell one-way ANOVA test was used for data that did not satisfy the test for homogeneity of variances. Pearson correlation analysis was performed for the relationship between variables. The enumeration data were tested using a χ^2^ test. The χ^2^ segmentation method was used for the pairwise comparison of the three groups of categorical variables (the test level *a* = 0.017 needed to be adjusted). Statistical results were analyzed using a two-sided *a* = 0.05, with *P* < 0.05 considered statistically significant.

## Results

### Peritoneal Cancer Index, Standardized Uptake Maximum Value, and Retention Index in Patients With Colorectal Peritoneal Carcinomatosis

**Figure 2:**
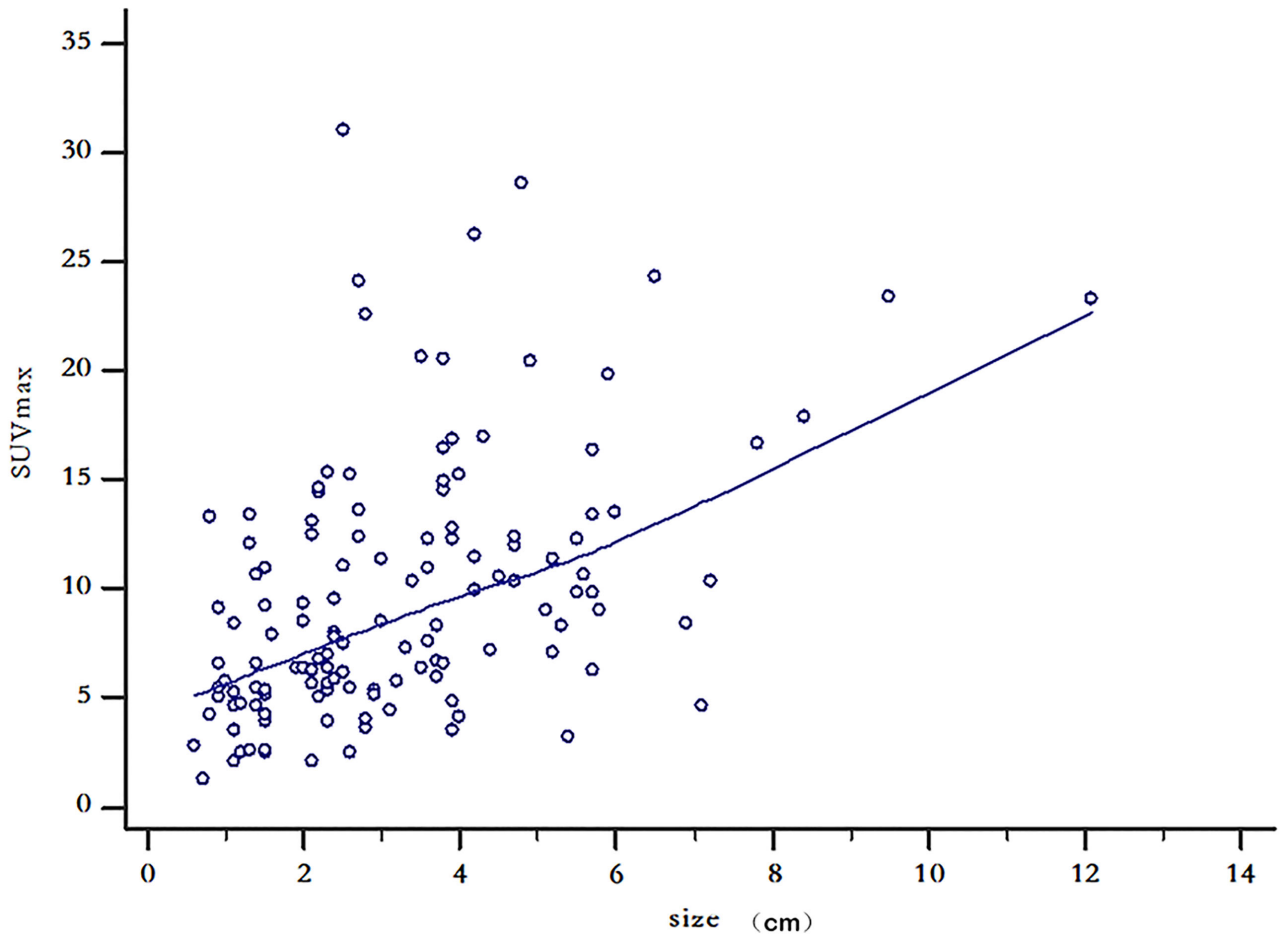
Scatter diagram of maximum lesion size and standardized uptake maximum value in 132 patients with colorectal peritoneal carcinomatosis.

The PCI range of the 132 patients with CRPC was 2–30, the mean was 7.40 ± 8.14, and the quartile was 2–8. The long diameter of the largest CRPC lesions in the 132 patients with CRC ranged from 0.6 to 12.1 cm, with an average of 3.23 ± 1.94 cm and quartiles of 1.83–4.20 cm. The SUV_max_ range of the lesions in patients with CRPC was 1.2 to 31.0, with a mean SUV_max_ of 9.65 ± 6.01 and quartiles of 5.30–12.33. The correlation coefficient between SUV_max_ and the size of the largest CRPC lesion was *r* = 0.47, with a 95% confidence interval (CI) of 0.33–0.59; this was statistically significant (*P* < 0.001). The scatter diagram is shown in **Figure 2**.

**Figure 3:**
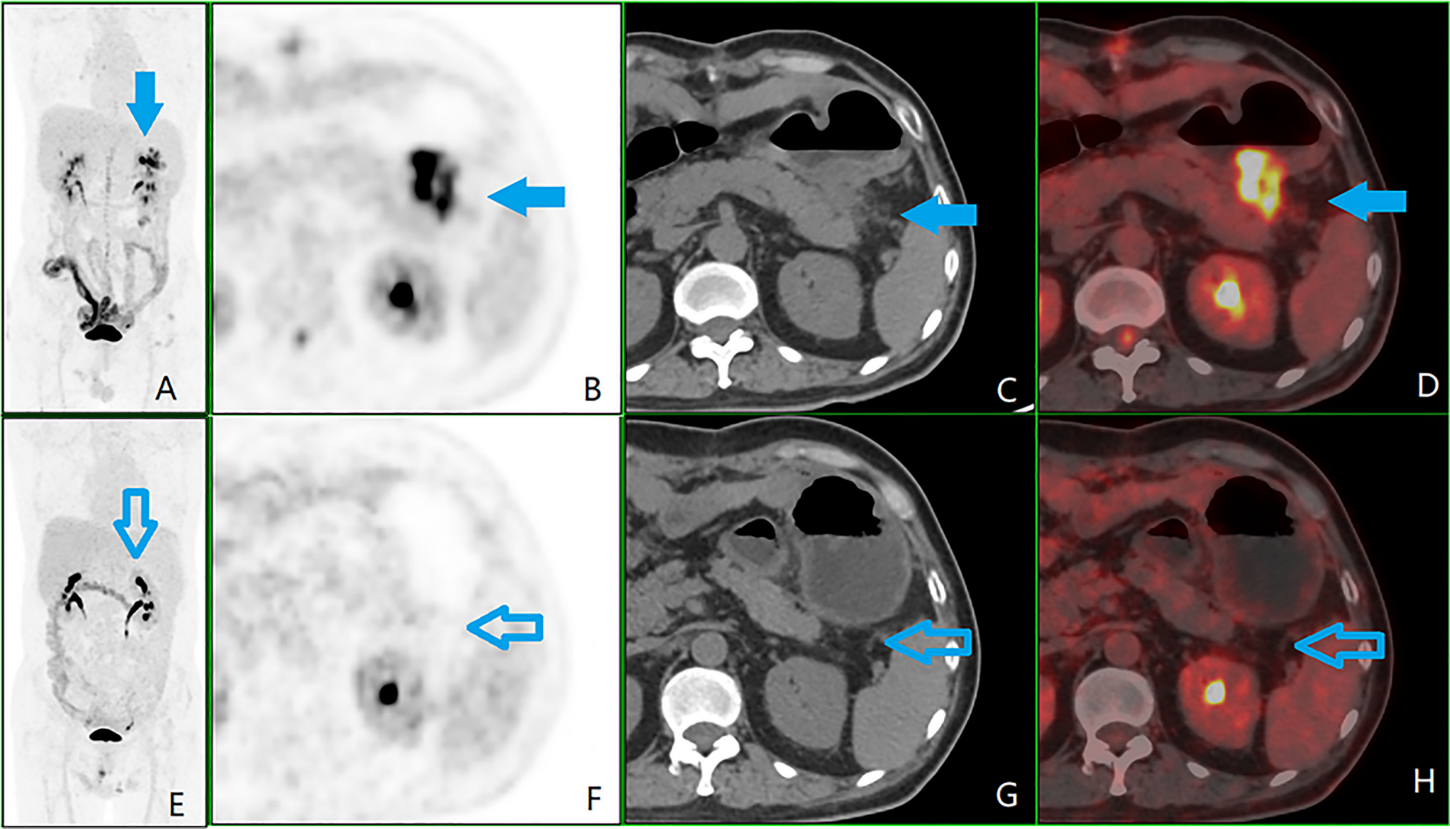
A patient with colorectal peritoneal carcinomatosis (CRPC), male, 56 years old, operated on for ulcerative poorly differentiated adenocarcinoma of the descending colon on May 17, 2017. **(A)** Positron emission tomography (PET) 3D-MIP image; **(B)** PET cross-sectional image; **(C)** computed tomography (CT) cross-sectional image; **(D)** PET/CT fusion cross-sectional image; **(E–H)** and **(A–D)** are in the same order, which is of the second ^18^F-fluorodeoxyglucose PET/CT examination on September 11, 2018, after six courses of chemotherapy. The first examination showed plaques with soft tissue density, i.e., CRPC lesions (shown by the blue arrow) in the left upper abdominal region (zone 3) next to the anterior tail of the pancreas, lesion size 2, with a peritoneal cancer index score of 2. The glucose metabolism was significantly elevated, with a standardized uptake maximum value of 20.5, a time-lapse period of 31.8, and a retention index of 55.1%. A review after completion of six courses of chemotherapy showed a significantly reduced, undefined CRPC lesion (shown by the hollow blue arrow).

A time-lapse examination was performed in 72 cases, with an RI range of -10.0%–112.2% and RI quartiles of 13.5%–48.9%. An RI ≥5% was positive in 65 cases, of which 43 had a significant increase in RI ≥20% (see **Figure 3**), and 22 had an increase in RI 5%–20%. There were seven cases with no significant change in RI <5% (negative).

### Differences in the Pathological Type of Colorectal Cancer, Peritoneal Cancer Index, Standardized Uptake Maximum Value, and Retention Index of Peritoneal Carcinomatosis in Different Locations of Colorectal Cancer

**Figure 4:**
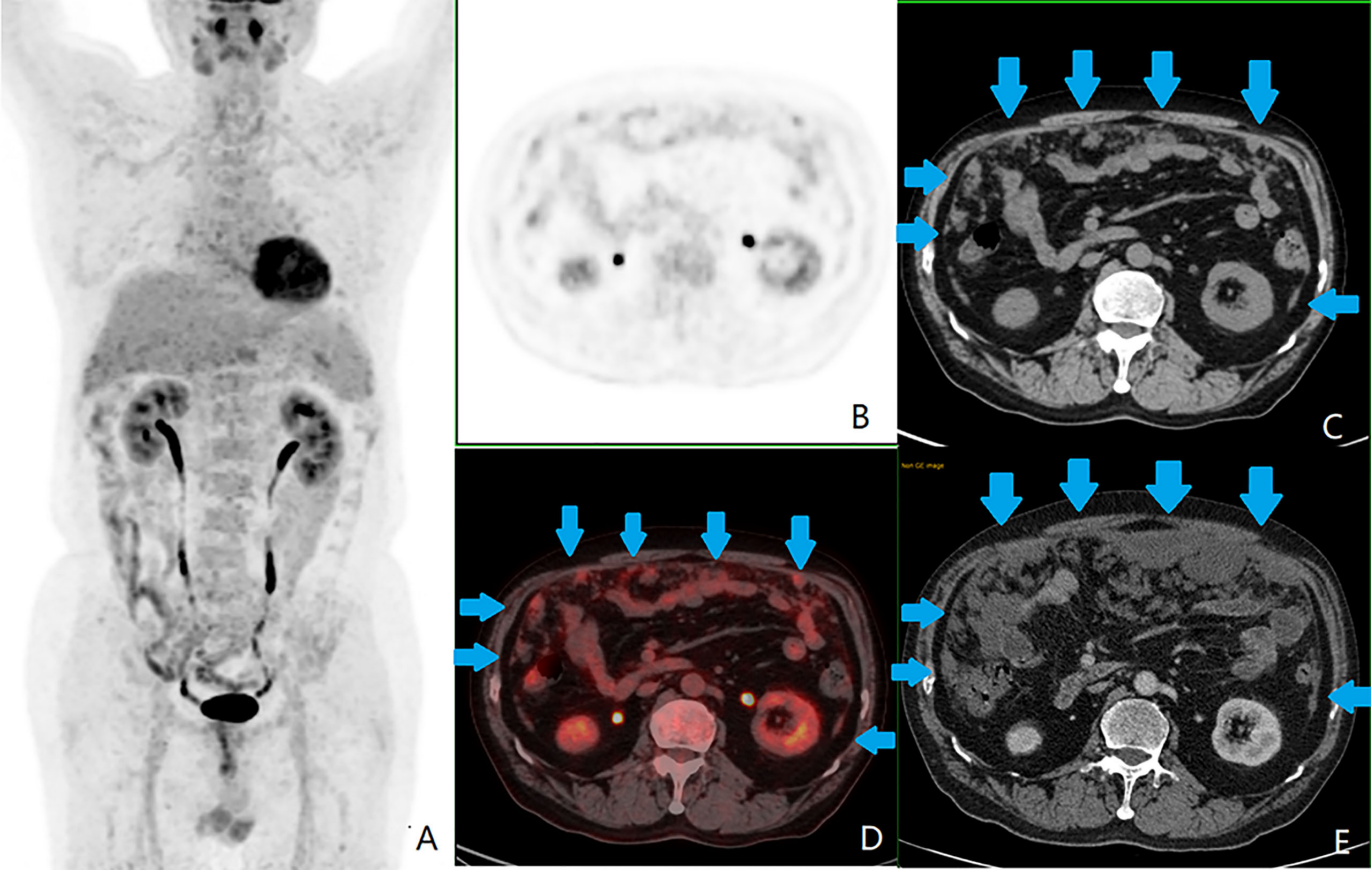
A patient with colorectal peritoneal carcinomatosis (CRPC), male, 68 years old, with mucinous adenocarcinoma of the appendix. **(A)** The positron emission tomography (PET) 3D-MIP image; **(B)** PET cross-sectional image; **(C, E)** computed tomography (CT) cross-sectional image; **(D)** PET/CT fusion cross-sectional image; **(A–D)** the first ^18^F-fluorodeoxyglucose PET/CT examination on October 11, 2016, which showed CRPC distributed in zones 0 ([lesion size] LS-2), 1 (LS-2), 2 (LS-2), 3 (LS-2), 4 (LS-2), 5 (LS-2), 6 (LS-2), 7 (LS-2), and 8 (LS-2), as shown by the blue arrow in images **(C, D)**. This patient had a peritoneal cancer index (PCI) score of 18. The largest lesion was located in the umbilical region (zone 0), measuring approximately 3.9 cm, with a standardized uptake maximum value of 4.8 g/ml with a small amount of seroperitoneum. **(E)** Contrast-enhanced abdominal and pelvic CT examination on September 14, 2019, showed significant progression of CRPC, with a PCI score of 26.

The cases were divided into three groups according to the location of the primary CRC: the RCC, LCC, and RC groups. The differences between the groups in the three observed indicators (pathological type of primary lesion, PCI, and RI) were statistically significant (*P* < 0.05), but the difference between the groups for SUV_max_ was not statistically significant (*P* > 0.05; see **Figures 3**, **4**). Detailed results are shown in **Table 2**. The three statistically significant observation indicators were then compared in pairs.

**Table 2:** Differences in pathological type of CRC, PCI, SUV_max_ and RI of peritoneal carcinomatosis in different locations of CRC.

		RCC group (37 cases)	LCC group (44 cases)	RC group (51 cases)	Statistics (χ^2^, F)	P value
**Pathological types^#^**	**Group A**	11	16	30	13.90	0.008*
	**Group B**	6	7	3		
	**Group C**	9	3	3		
**PCI**		9.84±9.41	8.16±8.79	4.98±5.82	4.28	0.02*
**SUV_max_**		8.64±5.81	10.05±5.59	10.04±6.50	0.72	0.49
**RI^&^**		0.13±0.11	0.37±0.20	0.37±0.30	14.06	0.001*

RCC group, LCC group and RC group refer to the right-sided colon cancer group, left-sided colon cancer group and rectal cancer group respectively; Group A, Group B, Group C refer to the moderately-well differentiated adenocarcinoma, moorly differentiated adenocarcinoma, mucinous adenocarcinoma. ^#^A total of 88 post-operative pathological analyses were performed, patients with adenocarcinoma as the gross pathological type were not included in the statistics; ^&^Seventy-two of 132 patients with CRPC underwent time-lapse imaging, 17, 23 and 32 in the RCC, LCC and RC groups, respectively. *P <0.05, indicating a statistically significant difference.

In terms of pathological type, the difference between the RCC and RC groups (χ^2^ = 9.49, *P* = 0.009) was statistically significant (*P* < 0.017). Mucinous and poorly differentiated adenocarcinomas were predominant in the RCC group, with mucinous adenocarcinomas being more common in the RCC group than in the RC group. The RC group was dominated by moderately well-differentiated adenocarcinomas. There was no statistically significant difference (*P* > 0.017) between the RCC and LCC groups (χ^2^ = 6.33, *P* = 0.04) and between the LCC and RC groups (χ^2^ = 2.22, *P* = 0.33) in pathological type.

For PCI, the difference between the RCC and RC groups (*P* = 0.02) was statistically significant, with the RCC group having a higher PCI than the RC group; the differences between the RCC and LCC groups (*P* = 0.69) and between the LCC and RC groups (*P* = 0.11) were not statistically significant.

For RI, a total of 72 patients with CRPC underwent time-lapse scanning, with statistically significant differences between the RCC and LCC groups (*t* = 4.88, *P* < 0.001) and between the RCC and RC groups (*t* = 3.94, *P* < 0.001), with lower RI in the RCC group than in the LCC and RC groups. The difference between the LCC and RC groups (*t* = 0.12, *P* = 0.90) was not statistically significant.

### Differences in Colorectal Cancer Location, Peritoneal Cancer Index, Standardized Uptake Maximum Value, and Retention Index of Peritoneal Carcinomatosis With Different Pathological Types of Colorectal Cancer

The cases were divided into three groups according to the pathological type of primary CRC lesion: the moderately well-differentiated, poorly differentiated, and mucinous adenocarcinoma groups. The difference between the three groups in the CRC location was statistically significant (*P* < 0.05), while the differences between the groups in the rest of the observed indexes (PCI, SUV_max_, and RI) were not statistically significant (*P* > 0.05; see **Table 3**).

**Table 3:** Differences in CRC location, PCI, SUV_max_ and RI of peritoneal carcinomatosis with different pathological types of CRC (88 cases).

		Group A (57 cases)	Group B (16 cases)	Group C (15 cases)	Statistics (χ^2^, F)	P value
**CRC location**	**RCC group**	11	6	9	13.90	0.01*
	**LCC group**	16	7	3		
	**RC group**	30	3	3		
**PCI**		5.88±6.79	9.19±8.54	9.67±8.41	2.31	0.11
**SUV_max_**		10.25±6.08	10.04±5.94	6.62±3.36	2.47	0.09
**RI^#^**		0.33±0.28	0.34±0.21	0.17±0.19	1.69	0.20

Group A, Group B, Group C refer to the moderately-well differentiated adenocarcinoma, poorly differentiated adenocarcinoma, mucinous adenocarcinoma. RCC group, LCC group and RC group refer to the right-sided colon cancer group, left-sided colon cancer group and rectal cancer group respectively; ^#^Time-lapse imaging was performed in 54 cases of 88 CRPC patients, 36 cases, 8 cases and 10 cases for the moderately-well differentiated adenocarcinoma, the poorly differentiated adenocarcinoma, and the mucinous adenocarcinoma group, respectively; *P <0.05, indicating a statistically significant difference.

Regarding the location of the primary CRC lesion, the difference between the moderately well-differentiated adenocarcinoma and mucinous adenocarcinoma groups was statistically significant (*P* < 0.017) (χ^2^ = 11.49, *P* = 0.003), with moderately well-differentiated adenocarcinomas predominant in the rectum and the mucinous adenocarcinomas predominant in the right-sided colon (see **Figure 4**). There was no statistically significant difference between the moderately well-differentiated adenocarcinoma and poorly differentiated adenocarcinoma groups (χ^2^ = 4.00, *P* = 0.14) and between the poorly differentiated adenocarcinoma and mucinous adenocarcinoma groups (χ^2^ = 4.36, *P* = 0.14) (*P* > 0.017).

As SUV_max_ was the most commonly used metabolic parameter, and the difference between the three groups was close to statistically significant (*P* = 0.09), further two-by-two comparisons showed that the SUV_max_ was higher in the moderately well-differentiated adenocarcinoma group than in the mucinous adenocarcinoma group. The difference between the two groups was statistically significant (*P* = 0.03). There was no statistically significant difference (*P* > 0.05) between the moderately well-differentiated adenocarcinoma and poorly differentiated adenocarcinoma groups and between the poorly differentiated adenocarcinoma and mucinous adenocarcinoma groups, as shown in **Figures 3**, **4**.

## Discussion

The present study showed a predominantly middle-aged and older population of patients with CRC with a high incidence of CRPC. The SUV_max_ of the CRPC lesions was positively correlated with their size. The most common pathological types in RCC were poorly differentiated and mucinous adenocarcinomas, while the most common type in RC were moderately well-differentiated adenocarcinomas. The extent and degree of involvement of peritoneal metastases in RCC were higher than in RC, but the SUV_max_ of peritoneal metastases in RCC was lower than in RC, and the RI was lower than in LCC and RC.

It has long been recognized that there are developmental and physiological differences between the anatomical parts of the colorectum. In recent years, the differences between RCC and LCC have become a focus of interest due to their different outcomes, prognoses, and clinical responses to chemotherapy (14). According to the American Cancer Center, trends in CRC incidence vary by site, with a gradual decline in LCC and an increase in RCC incidences of approximately 25% (15). Bufill (16) concluded that, because of the differences in clinicopathological features, diagnostic and treatment guidelines, and prognoses between LCC and RCC, colon cancer should be differentiated using the splenic flexure of the colon as the boundary, and RCC and LCC should be treated as two different diseases. Benedix et al. (17) concluded that the degree of tumor differentiation is closely related to a tumor’s location: the closer a tumor is to the ileocecal region, the less differentiated it is, and the closer it is to the sigmoid colon, the more differentiated it is. The same study also found that RCC lesions are generally larger and more advanced than LCC lesions, with the main histological features being poorly differentiated, mucinous-like indolent cells (18).

The RCC group in the present study differed from the RC group in terms of pathological type. The analysis suggested that there were 15 mucinous and poorly differentiated adenocarcinomas and 11 moderately well-differentiated adenocarcinomas in the RCC group, whereas there were six mucinous and poorly differentiated adenocarcinomas and 30 moderately well-differentiated adenocarcinomas in the RC group. There was a higher proportion of poorly differentiated tumors closer to the ileocecal region and a higher proportion of moderately well-differentiated tumors closer to the rectum, which is consistent with the results of previous studies (17, 18).

There is a consensus, both nationally and internationally, that peritoneal metastasis is a complex process involving multiple stages, factors, and molecular mechanisms. The intraperitoneal dissemination of CRC forms CRPC through direct spread, implant metastasis, surgical operation, trauma, etc. (19). In the present study, although there was some tumor heterogeneity, the nature of the primary tumor was often similar to that of the metastatic lesion. Compared with LCC, RCC more commonly involved mucinous adenocarcinoma and signet-ring cell carcinoma, which was less differentiated and more aggressive in its biological behavior (20). Related studies (21–23) have also shown that patients with mucinous adenocarcinoma have a significantly increased chance of developing CRPC. In the present study, the PCI scores in the RCC group were 9.84 ± 9.41, compared with 4.98 ± 5.82 in the RC group. The difference between the two groups was statistically significant, suggesting that the extent of peritoneal involvement was more extensive and severe in the RCC group than in the RC group. However, the results of the present study failed to show the difference between the RCC and LCC groups in terms of PCI and the pathological type of the primary lesion, which might be related to the small number of cases enrolled; further study of this aspect is therefore required.

Huang et al. (24) retrospectively analyzed the CT and PET/CT imaging data of 37 pathologically confirmed mucinous adenocarcinomas and 50 non-mucinous adenocarcinomas, finding the density and CT enhancement on plain and enhanced CT were significantly lower in the mucinous adenocarcinoma group than in the non-mucinous adenocarcinoma group. The degree of enhancement of hypodense areas within the lesion, the proportion of hypodense areas, and the proportion of lymph nodes and distant metastases were all higher in the mucinous adenocarcinoma group than in the non-mucinous adenocarcinoma group and the SUV_max_ was significantly lower in the mucinous adenocarcinoma group than in the non-mucinous adenocarcinoma group on PET/CT. Mucinous adenocarcinomas and signet-ring cell carcinomas had a large amount of mucin and mucus components with sparse vascularity, thus resulting in low density and low ^18^F-FDG metabolism on CT.

In the present study, the SUV_max_ of CRPC in the moderately well-differentiated adenocarcinoma group was 10.25 ± 6.08 g/ml, while the SUV_max_ of the mucinous adenocarcinoma group was 6.62 ± 3.36 g/ml. The difference between the two groups was statistically significant, showing that the level of ^18^F-FDG metabolism in CRPC in the mucinous adenocarcinoma group was generally lower than in the moderately well-differentiated adenocarcinoma group. In addition, as mucinous and poorly differentiated adenocarcinomas were predominant in the RCC group in the present study, whereas moderately well-differentiated adenocarcinomas were predominant in the LCC and RC groups, the RI in the RCC group was significantly lower than in the LCC and RC groups, suggesting that the increase in ^18^F-FDG metabolic levels in mucinous adenocarcinomas was limited and generally lower than in the non-mucinous adenocarcinoma group, even if the imaging time was increased. However, CRPC lesions with low RI might suggest a predominance of mucinous adenocarcinoma. When analyzing the PET/CT images of such patients, more emphasis needs to be placed on careful CT-based analysis of the anatomy and lesions rather than relying too heavily on significant PET-based markers of high glucose uptake.

The present study had some limitations. First, 44 patients with CRPC without postoperative pathology were excluded from further analysis, moderately well-differentiated adenocarcinomas were not studied in the subgroups, and the number of cases in the poorly differentiated and mucinous adenocarcinoma groups was low. Second, this study showed a positive correlation between SUV_max_ and the size of CRPC lesions and focused only on the largest lesions, but there were some sub-lesions with high SUV_max_, which might have led to some bias in the results. Third, the false-negative results in the imaging assessment of CRPC and the limitations of a single-center study could not be avoided. Future studies should work closely with relevant departments, such as the Department of Gastrointestinal Surgery and the Department of Oncology, to obtain complete pathological results for suspicious lesions. Furthermore, the sample size should be expanded for a more efficient prospective study of CRPC.

## Conclusion

The PCI, SUV_max_, and RI of peritoneal metastatic carcinoma caused by CRC in varying locations and pathological types are different. Mucinous and poorly differentiated adenocarcinomas are fairly common in the right colon, and the PCI of peritoneal metastatic carcinoma is rather high but with relatively lower glucose metabolism indexes (SUV_max_ and RI). The SUV_max_ of CRPC lesions is positively correlated with size. The use of ^18^F-FDG PET/CT, especially glucose metabolism functional imaging, facilitates the detection of CRPC in moderately well-differentiated adenocarcinomas, and it is of great clinical value and significance for diagnosing and evaluating CRPC in patients with CRC.

## Data Availability Statement

The original contributions presented in the study are included in the article/supplementary material. Further inquiries can be directed to the corresponding author.

## Ethics Statement

The studies involving human participants were reviewed and approved by Affiliated Hospital of Nantong University. The patients/participants provided their written informed consent to participate in this study.

## Author Contributions

Conception and design of the research: C-FS, CS. Acquisition of data: DZ, YG, X-YM. Analysis and interpretation of the data: C-FS, Z-HT. Statistical analysis: S-LB. Obtaining financing: C-FS, CS. Writing of the manuscript: C-FS, DZ. Critical revision of the manuscript for intellectual content: C-FS, CS. All authors read and approved the final draft. All authors contributed to the article and approved the submitted version.

## Funding

Scientific research project on elderly health of Jiangsu Health Commission (LK2021019); Nantong Social Livelihood Science and Technology Project (MS12020043); Nantong “14th Five-Year” Science and Education Strengthening Health Project Young Medical Talents Project (2021-2025); Affiliated Hospital of Nantong University Scientific Research Talents Training Project Angel Program (2020).

## Conflict of Interest

The authors declare that the research was conducted in the absence of any commercial or financial relationships that could be construed as a potential conflict of interest.

## Publisher’s Note

All claims expressed in this article are solely those of the authors and do not necessarily represent those of their affiliated organizations, or those of the publisher, the editors and the reviewers. Any product that may be evaluated in this article, or claim that may be made by its manufacturer, is not guaranteed or endorsed by the publisher.
